# MineBL: A Battery-Free Localization Scheme with Binocular Camera for Coal Mine

**DOI:** 10.3390/s22176511

**Published:** 2022-08-29

**Authors:** Song Qu, Zhongxu Bao, Yuqing Yin, Xu Yang

**Affiliations:** 1 School of Computer Science and Technology, China University of Mining and Technology, Xuzhou 221000, China; 2 Technical Department, Xuzhou Kerui Mining Technology Co., Ltd., Xuzhou 221000, China

**Keywords:** localization, coal mine, battery-free

## Abstract

Accurate localization in underground coal mining is a challenging technology in coal mine safety production. This paper proposes a low-cost battery-free localization scheme based on depth images, called MineBL. The main idea is to utilize the battery-free low-cost reflective balls as position nodes and realize underground target localization with a series of algorithms. In particular, the paper designs a data enhancement strategy based on small-target reorganization to increase the identification accuracy of tiny position nodes. Moreover, a novel ranging algorithm based on multi-filter cooperative denoising has been proposed, and an optimized weighted centroid location algorithm based on multilateral location errors has been designed to minimize underground localization errors. Many experiments in the indoor laboratories and the underground coal mine laboratories have been conducted, and the experimental results have verified that MineBL has good localization performances, with localization errors less than 30 cm in 95% of cases. Therefore, MineBL has great potential to provide a low-cost and effective solution for precise target localization in complex underground environments.

## 1. Introduction

Coal—as one of the energy mineral resources—occupies an important position in the production and consumption of primary energy. However, due to the harsh underground mining environment and the constantly changing working face, it is particularly crucial to ensure the safety of underground personnel and production efficiency effectively [1]. The localization technology in underground coal mines plays a prominent role in safe mining, personnel monitoring and scheduling, and underground post-disaster rescue [2,3]. Due to the complex underground environments and technical challenges, the wireless localization effect of underground targets is unsatisfactory [4,5]. Therefore, achieving the precise localization of underground targets has always been important to the safe production and intelligent construction of coal mines.

Existing underground localization technologies mainly include Wi-Fi [6,7,8,9], Bluetooth [10,11,12], UWB [13,14,15], Zigbee [16], RFID [17,18], as well as infrared technology [19,20,21]. However, there are many limitations in the practical application of the above technologies in the underground coal mines. (1) Security risk issues: The above localization scheme needs to deploy active localization base stations underground in advance, and such base stations usually require an external power supply, which is prone to safety accidents. (2) Low localization accuracy issues: Harsh underground coal mine environments as well as complex and changeable tunnel structures, lead to significant multipath effects and severe signal attenuations of wireless signals, resulting in low location accuracy of wireless signal-based underground localization techniques [22,23,24]. (3) High deployment cost issues: The deployment of active localization base stations based on the above underground localization techniques has high costs/maintenance costs, which widely hinder applications in large-scale underground coal mines [25,26,27].

The underground localization technique based on vision sensors has become a recent research hotspot for several reasons, e.g., no electromagnetic interference and low cost [28]. This technology achieves underground target localization by identifying position nodes in images and obtaining location information of the nodes [29,30,31,32]. However, the traditional visual localization techniques based on monocular [33,34] or binocular cameras [35] are easily influenced by unfavorable illumination conditions in the underground, resulting in low accuracy of underground target recognition and large distance calculation errors. Compared to monocular or binocular cameras, depth-sensing cameras can carry out active ranging and they are robust to dark environments [36,37,38]; they have good application prospects in underground coal mines. Nevertheless, most of the depth-image based localization techniques need to locate the target carrying a camera [39], which may incur high deployment costs due to the high mobility of underground personnel and equipment. Moreover, some localization schemes use fixed cameras for locating [40]. However, due to multiple production processes and frequent changes in the working face, such localization solutions require fixed cameras to be deployed several m apart or cameras to be redeployed as the working face moves. The significant deployment and maintenance costs may limit their applications in underground environments. Therefore, a low-cost and easily deployed battery-free underground localization system is urgently needed.

**Figure 1:**
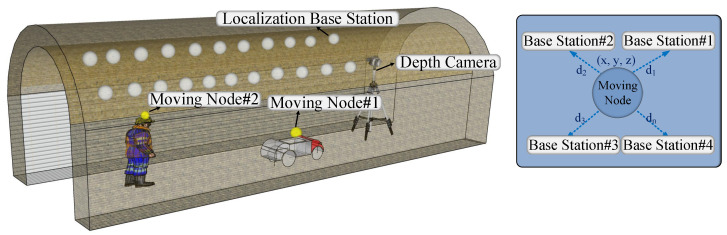
MineBL scene.

To solve the above problems, this paper proposes a low-cost battery-free localization based on depth images in underground coal mines, called MineBL. The low-cost battery-free reflective balls are utilized as position nodes to realize the underground target localization by combining a series of localization algorithms. Figure 1 illustrates the main ideas of the localization method proposed in this paper. A depth camera is introduced to identify the position nodes, and then the nodes with known coordinates are taken as the base stations, while those carried by the localization targets are regarded as the unknown nodes. The distance between the base stations and the unknown nodes is measured to obtain the location information of the localization targets. However, many challenges still exist when implementing this localization scheme, mainly in the following aspects. Firstly, the complex underground environments make the system extremely difficult to recognize the deployed tiny position nodes. Secondly, the depth images collected by the depth cameras often contain a lot of noise, which may make the range accuracy of the system unstable. Thirdly, the accuracies of the traditional multilateral localization methods highly depend on the range accuracy, leading to the low localization accuracy of the system. In order to solve the first challenge, this paper proposes a data enhancement strategy based on small-target reorganization and further designs a micro-position node recognition algorithm for complex underground environments. For the second challenge, a multi-filter cooperative denoising algorithm was designed to obtain the relative distance between the base stations and targets. For the third challenge, we present a weighted centroid localization algorithm based on multi-location errors, which determine the weighting factors according to the error size of every location data. To summarize, the main contributions of this paper are as follows.

- The paper proposes an underground battery-free localization scheme called MineBL. To the best of our knowledge, it is the first paper to propose a low-cost battery-free localization scheme based on depth images in an underground coal mine, which can realize the accurate and safe localization of underground targets. MineBL can be deployed on a wide range of mobile devices, such as inspection robots, which has good mobility and can migrate with underground working faces.
- The paper also proposes a novel range algorithm based on collaborative denoising of multiple filters and has designed an optimized weighted centroid localization algorithm based on multilateral location errors to minimize underground location errors. The above methods can be applied to other underground localization systems.
- A large number of experiments in the indoor laboratories and the underground coal mine laboratories have been conducted, and the experimental results have verified that the MineBL underground localization scheme has good localization performances, with localization errors less than 30 cm in 95% of cases.

## 2. System Overview

**Figure 2:**
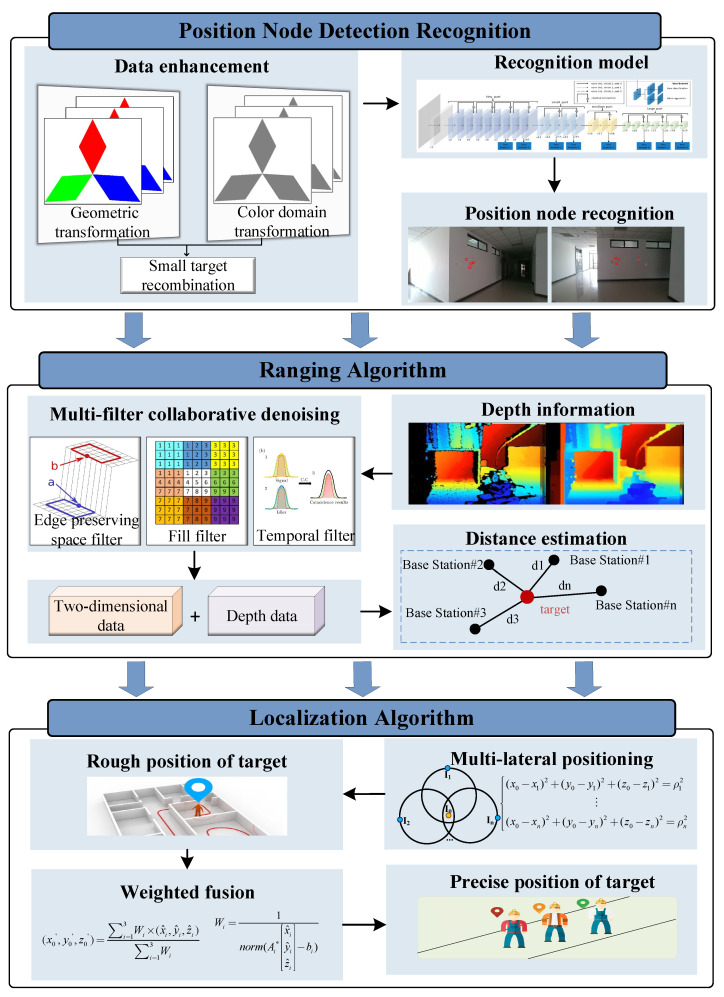
System overview.

This section provides a detailed overview of the overall MineBL framework. MineBL devices include position nodes (base stations and unknown nodes), depth cameras, and an edge computing node. As shown in Figure 2, localization base stations with known coordinates are prepositioned underground, and the targets with unknown nodes move underground. The position node recognition algorithm identifies the position nodes from images captured by depth cameras through deep learning methods. In particular, the data enhancement strategy of small-target reorganization is proposed to increase the recognition accuracy of small position nodes in complex coal mine environments. Based on the multi-filter collaborative denoising algorithm, the depth information is denoised to obtain accurate information on depth, and the relative distance between base stations and the targets is calculated by combining the two-dimensional information. Finally, an optimized centroid algorithm based on multilateral localization is proposed, which takes the reciprocal of coordinate errors as the weight and integrates the localization results of multiple sets of data to obtain the location of the targets accurately.

## 3. System Design

### 3.1. Position Node Recognition

It is difficult to detect tiny position nodes in complex coal mine environments. On the one hand, small targets contain fewer pixels and have little influence on the overall loss function of the images. On the other hand, small targets are prone to be mismarked and missed. To solve these problems, this paper proposes a data enhancement strategy based on small-target reorganization to enhance the recognition accuracy of position nodes. Then, the YOLOV5 model is used to identify the position nodes in the images.

#### 3.1.1. Data Collection

Position node images were collected from indoor corridors and mines with different angles, different distances, and different light intensities, respectively. A total of 2200 images were collected for the dataset—1100 images of position nodes under the indoor corridor and 1100 images of position nodes under the indoor corridor. For the collected data sets, annotation software was used to label the position nodes of each image, and the labeled data sets were divided into the training set and test set, with 1760 training sets and 440 test sets in total.

#### 3.1.2. Data Enhancement

To enhance the robustness of the model, a data augmentation strategy was used for the training dataset. Firstly, basic data enhancement techniques, such as geometric transformation and color domain transformation, were employed to expand the dataset. Geometric transformation enhancement refers to the geometric transformation of the images, including flipping, rotation, cropping, deformation, scaling, random cropping, and other operations [41], to reduce the impact of the camera angle as well as the distance of the object from the camera on the final recognition results. The data enhancement of color transformation changes the content of the images, so common conducts include adjusting brightness or saturation and noise injection [42]. Randomly adjusting the brightness or saturation of an image within a certain range can make the data set suitable for different lighting conditions. Noise injection involves injecting normal distributed Gaussian noise into the images so that the model can learn more stable features of the dataset.

Although the above data enhancement methods expand the training data to a certain extent, the number of positive samples of small targets is much smaller than that of large targets, which still leads to the model training imbalance [43]. To solve this problem, this paper proposes a data enhancement algorithm based on small-target reorganization, including the parts of copy–paste and histogram matching, which can enhance the recognition accuracy of position nodes by increasing the frequency of the position node occurrence in the images. Specifically, the copy–paste method is used to generate more position nodes and increase the number of position nodes in images, as well as the number of images containing position nodes in order to make the training more balanced. Meanwhile, under the condition of diversified illumination levels between images, if the position nodes of an image are simply inserted into another image, the added position nodes will not match the background and introduce a lot of noise into the newly generated images. Therefore, the paper introduces the histogram matching method of images on the basis of the copy–paste strategy. Histogram matching means that the histogram of one image is registered according to the histogram of the other image so that the histogram distribution of the two images is consistent. Before the “copy–paste” of the position nodes, an image of the position nodes is histogram-matched with the other to be inserted so that the position nodes are better integrated into the images to be inserted.

#### 3.1.3. Recognition Model

**Figure 3:**
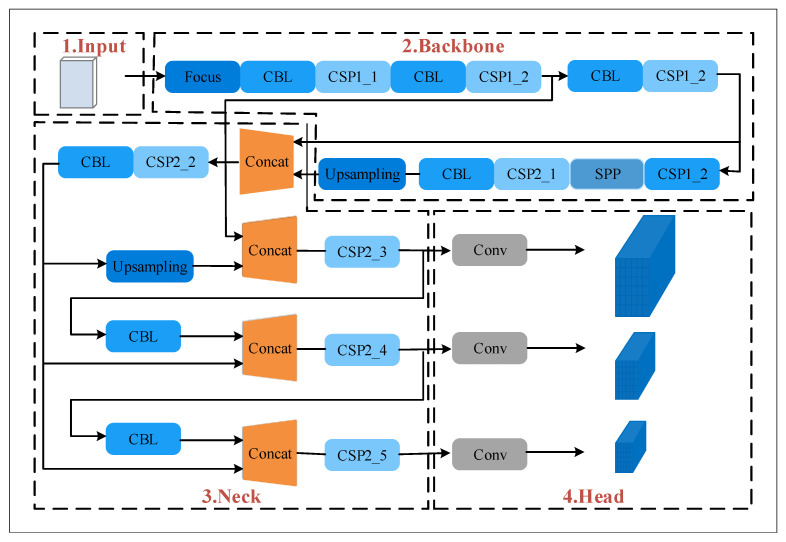
Position node recognition model.

Due to the different appearances, shapes, and postures of various objects, and the interference of illumination and occlusion, target recognition is the most challenging problem in computer vision. Th YOLO algorithm is one of the most popular algorithms in target recognition [44,45,46]; YOLOV5 was selected for the target recognition of position nodes in the paper. The position node detection model based on YOLOV5 is shown in Figure 3. The model is mainly composed of the focus module, convolution, and batch normalization (CBL) module, cross-stage partial (CSP) module, scaling cross-stage partial (SPP) module, standard convolution (Conv) module, and the tensor stitching (Concat) module. CSP1_x means that the CSP1 has *x* residual units, CSP2_x means that the CSP2 has 2x residual units, and *x* affects the depth of the network structure. The detection model is divided into the input, backbone, neck, and head according to the processing stages. Specifically, the input stage preprocesses the image, the backbone stage extracts feature information from the input image, the neck stage fuses the feature information extracted from the backbone part and the head stage calculates target category probability and position coordinates through loss functions to obtain target prediction results.

The recognition process of position nodes is as follows: First, the input image is divided into N×N cells, and each cell generates a prior box for the targets of different scales, such as large, medium, and small. If the center of the target is in a grid, the prior box of the grid is responsible for identifying the target. As shown in Equation (1), YOLOV5 uses the confidence degree *c* to represent the target classification probability and the performance of matching targets in the prior box.

(1) c=PH

where *P* is the target probability in the prediction box. If there is no target in the prediction box, *P* is 0, otherwise 1. *H* is the intersection ratio between the predicted box and the real box. Then, the segmented images are normalized, and the normalized dataset is sent to the lower layer feature extraction network for feature extraction. Next, the prediction box is set by k-means clustering, and the position of the prediction box is calculated. According to the offset value of the predicted coordinates, the location of the target center point and the width and height of the prediction box are calculated. Finally, the position node identification result is the output.

### 3.2. Ranging Algorithm

After the position nodes are detected by the deep learning model, the relative distance between the base stations and the localization targets can be obtained by using the information from depth images. However, many noises exist in the original information, so the localization accuracy is unsatisfactory. To solve this problem, the paper proposes a multi-filter collaborative denoising range algorithm. The noises in the original information of depth images are processed to obtain the exact absolute depth of position nodes firstly. Then the relative distance between base stations and the location targets can be acquired by combining the two-dimensional information.

#### 3.2.1. Multi-Filter Cooperative Denoising

Firstly, an edge-preserving spatial filter is adopted to remove noises while preserving edge details. For the depth images based on triangulation, the noises increase by a square as the distance from the objects to the cameras increase, resulting in excessive smooth edge information of the near images and insufficient smooth edge information of the distant images. Thus, converting the depth images back to disparity images preserves the edge details of the distant objects since disparity is approximately inversely proportional to depth.

**Figure 4:**
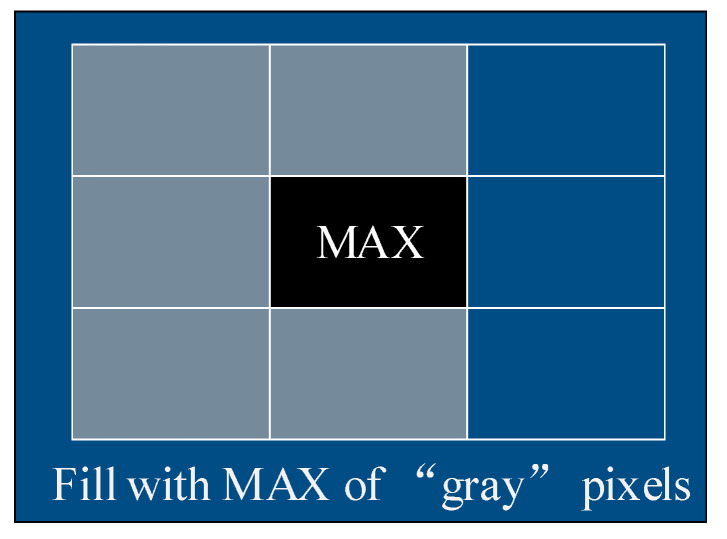
Fill filter.

Secondly, a filling filter is employed to fill the black spots in-depth images. As the main camera of depth adopted in the paper is the left camera, the number of objects seen by the left or the right cameras is usually inconsistent. Specifically, when the objects seen by the left camera have never appeared in the right camera, the depth of these locations cannot be calculated, which is reflected in-depth images as a black dot with a depth value of 0, due to the lack of disparity information. Moreover, the black dots also appear when overexposed or underexposed, or when objects are too close to the camera, less than the minimum detectable distance. As shown in Figure 4, the maximum (farthest) of “gray” pixels are selected in the paper to fill the black spot area.

Finally, a time filter is utilized to remove the noises caused by conditional changes. As the conditions may change, such as light changes, dynamic changes, and noises caused by the infrared emitters, the past apparent information may be lost, and excessive noises may be introduced, because there is no connection between the frame information provided by the cameras; that is, each frame is independent. Thus, the methods of the time filter and the exponential moving average are used to “remember” the depth value of a pixel in the previous frame number and take the average value. The exponential moving average, also known as weighted moving average, is an averaging method that gives higher weight to recent data. The EMA value at time *t* is shown as Equations (2).

(2) $$S_{t}=\left\{\begin{array}{cc} Y_{1}, & t=1\\ aY_{t}+\left(1-a\right)S_{t-1}, & t>1\;\mathrm{and}\;\Delta =\left|S_{t}-S_{t-1}\right|<\delta_{\mathrm{thresh}\;}\\ Y_{t}, & t>1\;\mathrm{and}\;\Delta =\left|S_{t}-S_{t-1}\right|>\delta_{\mathrm{thresh}\;} \end{array}\right.$$

where *a* represents the degree to which the weight is reduced, $Y_{t}$ represents instant disparity values or depth values, $S_{t-1}$ is the EMA value at time t−1, $\delta_{\mathrm{thresh}\;}$ is one of the custom setting param on behalf of the depth threshold of the adjacent pixels. If the threshold is exceeded, edges will be present and the smoothing effect of the time filter will be temporarily disabled to prevent the edges from being smoothed.

**Figure 5:**
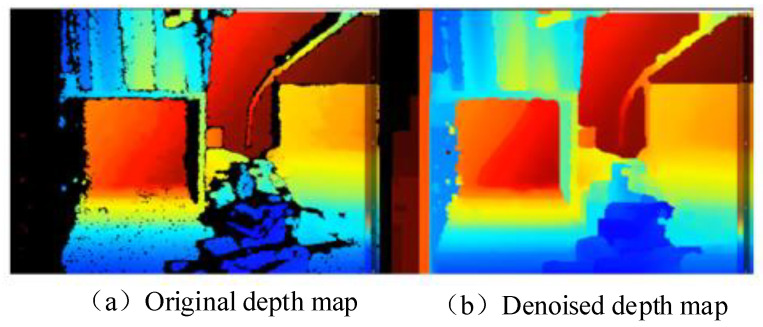
Denoising performance.

After the collaborative applications of the above three filters, the effect is shown in Figure 5. It can be seen that the quality of depth images has been improved significantly and the black holes have been significantly reduced, which can improve the range accuracy.

#### 3.2.2. Target-To-Base Station Ranging

**Figure 6:**
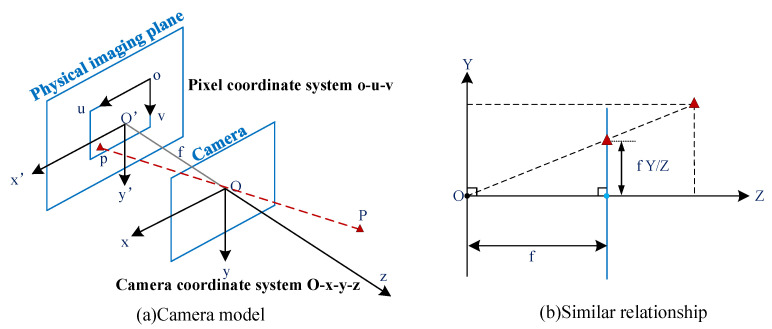
Camera model.

After obtaining the absolute depth of the position nodes, the relative distance between base stations and the targets can be obtained by combining the two-dimensional information. As shown in Figure 6, according to the simple camera model, 3D point P(X,Y,Z) is projected onto point p(u,v) on the 2D images, and has the relationship shown in Equation (3).

(3) p=K[R∣T]P

where *p* is a point in the 2D plane, *K* is the camera’s internal parameter matrix. [R|T] is the external parameter matrix, used to describe the Euclidean transformation between the world coordinate system and the camera coordinate system. *P* is [X,Y,Z,1], describing the world coordinate information of 3D points. The matrix form of Equation (3) is as follows.

(4) $$s\left[\begin{array}{c} u\\ v\\ 1 \end{array}\right]=\left[\begin{array}{ccc} f_{x} & 0 & u_{0}\\ 0 & f_{y} & v_{0}\\ 0 & 0 & 1 \end{array}\right]\left[\begin{array}{cccc} r_{11} & r_{12} & r_{13} & t_{1}\\ r_{21} & r_{22} & r_{23} & t_{2}\\ r_{31} & r_{32} & r_{33} & t_{3} \end{array}\right]\left[\begin{array}{c} X\\ Y\\ Z\\ 1 \end{array}\right]$$

where *s* is a scaling coefficient that varies with focal length. Without considering the interconversion between the world coordinates and the camera coordinates (that is, using the camera coordinate system), the *R* and *T* can be expressed as shown in Equation (5).

(5) $$R=\left[\begin{array}{ccc} r_{11} & r_{12} & r_{13}\\ r_{21} & r_{22} & r_{23}\\ r_{31} & r_{32} & r_{33} \end{array}\right]=\left[\begin{array}{ccc} 1 & 0 & 0\\ 0 & 1 & 0\\ 0 & 0 & 1 \end{array}\right],T=\left[\begin{array}{c} t_{1}\\ t_{2}\\ t_{3} \end{array}\right]=\left[\begin{array}{c} 0\\ 0\\ 0 \end{array}\right]$$

Substitute Equation (5) into Equation (4), as shown in Equations (6).

(6) $$\left\{\begin{array}{c} u=\frac{f_{x}X}{Z}+u_{0}\\ v=\frac{f_{y}Y}{Z}+v_{0} \end{array}\left\{\begin{array}{c} X=\frac{Z\left(u-u_{0}\right)}{f_{x}}\\ Y=\frac{Z\left(v-v_{0}\right)}{f_{y}}\\ Z=s \end{array}\right.\right.$$

where $f_{x}$ and $f_{y}$ are the focal lengths after scaling, while $u_{0}$ and $v_{0}$ are the numbers of translations at the origin. Then, the 2D and 3D coordinates can be converted to and from each other using the *Z* information provided by depth images.

After calculating the 3D coordinates of every position node in the camera coordinate system, the relative distance $\rho_{i}$ between the base station and the coordinate value $\left(x_{i},y_{i},z_{i}\right)$ and the target with the coordinate value $\left(x_{0},y_{0},z_{0}\right)$ can be calculated as shown in Equation (7).

(7) $$\rho_{i}=\sqrt{{\left(x_{0}-x_{i}\right)}^{2}+{\left(y_{0}-y_{i}\right)}^{2}+{\left(z_{0}-z_{i}\right)}^{2}}$$

### 3.3. Localization Algorithm

#### 3.3.1. Multilateral Localization Model

**Figure 7:**
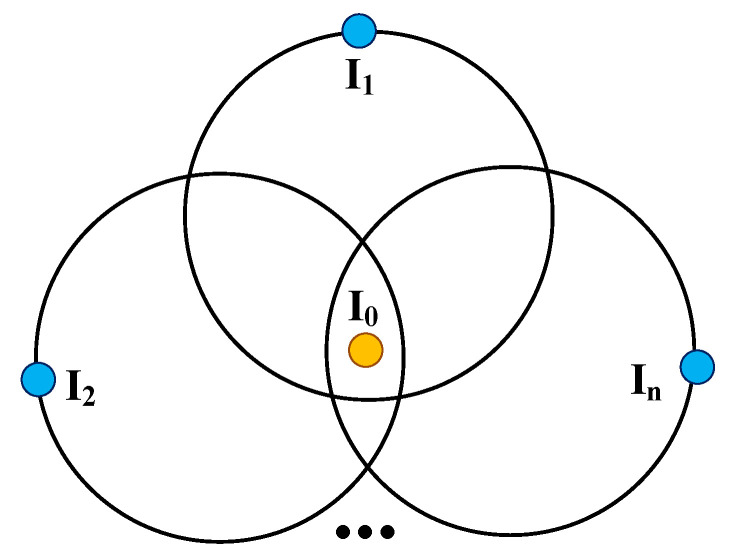
Multilateral localization.

The multilateral localization method is a common localization method in the localization system. As shown in Figure 7, suppose there were *n* localization base stations, $I_{1}$, $I_{2}$,..., $I_{n}$, with the coordinate values $\left(x_{1},y_{1},z_{1}\right)$, $\left(x_{1},y_{1},z_{1}\right)$,..., $\left(x_{n},y_{n},z_{n}\right)$, the distances from them to an unknown node $I_{0}\left(x_{0},y_{0},z_{0}\right)$ are $\rho_{1}$, $\rho_{2}$,..., $\rho_{n}$; Equations (8) can be deduced.

(8) $$\left\{\begin{array}{c} {\left(x_{0}-x_{1}\right)}^{2}+{\left(y_{0}-y_{1}\right)}^{2}+{\left(z_{0}-z_{1}\right)}^{2}=\rho_{1}^{2}\\ \vdots \\ {\left(x_{0}-x_{n}\right)}^{2}+{\left(y_{0}-y_{n}\right)}^{2}+{\left(z_{0}-z_{n}\right)}^{2}=\rho_{n}^{2} \end{array}\right.$$

As shown in Equation (9), Equation (8) can be expressed in matrix form.

(9) AX=L

where *A* is the coefficient matrix, and *L* is the constant vector. *X* is the unknown vector ${\left(x_{0},y_{0},z_{0}\right)}^{T}$. *A* and *L* can be expressed as Equations (10).

(10) $$\begin{array}{c} A=\left[\begin{array}{ccc} 2\left(x_{1}-x_{n}\right) & 2\left(y_{1}-y_{n}\right) & 2\left(z_{1}-z_{n}\right)\\ \vdots & \vdots & \vdots \\ 2\left(x_{n-1}-x_{n}\right) & 2\left(y_{n-1}-y_{n}\right) & 2\left(z_{n-1}-z_{n}\right) \end{array}\right]\\ L=\left[\begin{array}{c} \rho_{n}^{2}-\rho_{1}^{2}+x_{1}^{2}-x_{n}^{2}+y_{1}^{2}-y_{n}^{2}+z_{1}^{2}-z_{n}^{2}\\ \vdots \\ \rho_{n}^{2}-\rho_{n-1}^{2}+x_{n-1}^{2}-x_{n}^{2}+y_{n-1}^{2}-y_{n}^{2}+z_{n-1}^{2}-z_{n}^{2} \end{array}\right] \end{array}$$

As shown in Equation (11), the estimated coordinate value of the unknown node can be calculated by the least square method.

(11) $$X={\left(A^{T}A\right)}^{-1}L$$

As shown in Equation (11), when the error of *L* on the right side of the equation is small, the positioning effect is good and the estimated coordinate *X* is close to the real coordinate. However, in the actual positioning process, due to the environmental interference and other factors caused by inaccurate ranging, the error of *L* is large, and the *X* will seriously deviate from the true value of the unknown node. Therefore, the localization algorithm needs to be further improved.

#### 3.3.2. Traditional Centroid Algorithm

In the localization algorithm based on range, the errors of localization results with only one set of data may be huge, so it is necessary to synthesize multiple sets of data. In the traditional centroid algorithm, based on range-based localization with *N* sets of data, the average value of *N* estimated locations is generally taken as the final result. The traditional centroid algorithm is shown in the formula. The traditional centroid algorithm defaults to the equal weights of estimation coordinates of each group of data, which fails to reflect the differential impacts of the data.

#### 3.3.3. Weighted Centroid Algorithm

The paper proposes an improved weighted centroid algorithm based on a multilateral localization by synthesizing multiple sets of data. When solving the location coordinate equation, if *L* is the exact value, the accurate estimated coordinate value *X* can be obtained. At this point, the left and right sides of the equation AX=L are equal. Meanwhile, the greater the error of *L*, the greater the error of the least square solution equation, and the lower the reliability of data. Therefore, in the improved weighted centroid algorithm, the reciprocal of the coordinate error value is taken as the weight. The specific expressions of the algorithm are shown in Equation (12), Equation (13), and Equation (14).

(12) $$\left(x_{0}^{\prime },y_{0}^{\prime },z_{0}^{\prime }\right)=\frac{\sum_{i=1}^{3}W_{i}\times \left({\hat{x}}_{i},{\hat{y}}_{i},{\hat{z}}_{i}\right)}{\sum_{i=1}^{3}W_{i}}$$

(13) $$W_{i}=\frac{1}{\mathrm{norm}\left(A_{i}^{*}\left[\begin{array}{c} {\hat{x}}_{i}\\ {\hat{y}}_{i}\\ {\hat{z}}_{i} \end{array}\right]-b_{i}\right)}$$

(14) $$\mathrm{norm}\left(X\right)=\sqrt{\left({\left|X_{1}\right|}^{2}+{\left|X_{2}\right|}^{2}+\ldots +{\left|X_{n}\right|}^{2}\right)}$$

where $\left(x_{0}^{\prime },y_{0}^{\prime },z_{0}^{\prime }\right)$ represent the location coordinates of the targets determined by the weighted centroid algorithm and $W_{i}$ indicates the weights of each group of data. $\left({\hat{x}}_{i},{\hat{y}}_{i},{\hat{z}}_{i}\right)$ shows the coordinate value estimated by the multilateral localization algorithm of the data of group *i*, while $A_{i}$ and $L_{i}$ denote the linear equation param determined by the data of group *i*, and norm(X) expresses the binary norm of *X*.

## 4. Experiment and Evaluation

### 4.1. Simulation Results

Firstly, the simulation experiments were carried out to verify the feasibility of the experiments. The simulation experiments mainly evaluated the performances of the localization algorithm, and the influences of range errors and the number of localization base stations on the performances of the localization algorithms. The simulation area was 40 m long, 5 m wide, and 3 m high. Every setup ran 500 times and the average results are reported.

#### 4.1.1. Localization Performance

**Figure 8:**
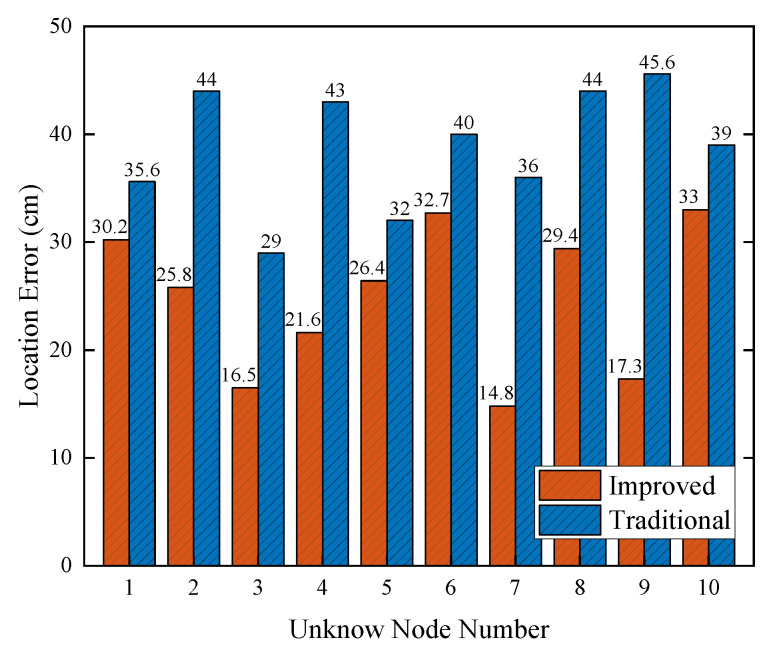
Overall localization performance.

Six localization base stations and 10 unknown nodes were deployed in the simulation area. When the standard deviation of ranging errors was 10 m, the simulation localization results of the traditional weighted centroid algorithm and the optimized weighted centroid algorithm in this paper are shown in Figure 8. It can be seen that the average localization error of the traditional weighted centroid algorithm was 34.4 cm and that of the algorithm described in this paper was 24.7 cm. Thus, the localization accuracy of the proposed algorithm in the paper is higher than that of the traditional weighted centroid algorithm.

#### 4.1.2. Impact of Range Error

**Figure 9:**
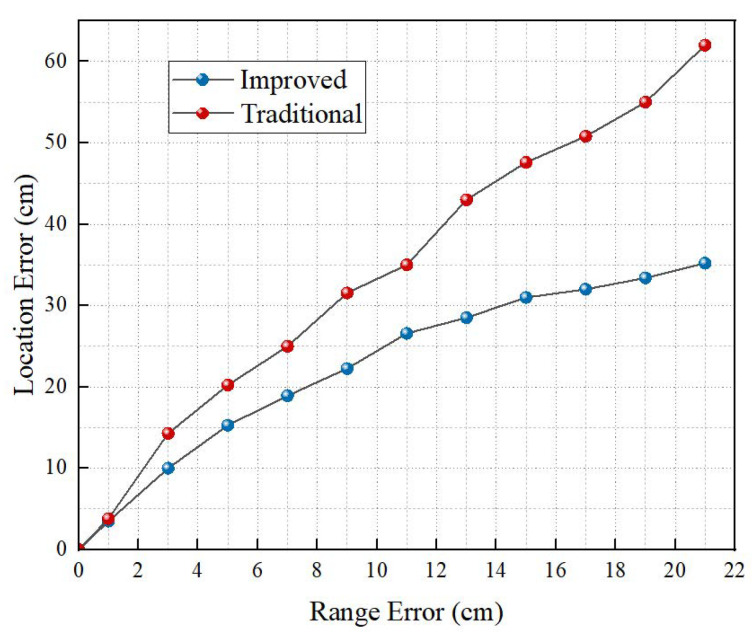
Impact of the range error on the localization performance.

This section evaluates the effects of range errors on localization accuracy. The range error was evenly measured between 0 and 21 cm. After large quantities of simulation experiments, the results are shown in Figure 9. It has been proven that the localization accuracy of the two algorithms is close when the range errors are small. With the increase of range errors, the localization errors of the two algorithms increase. However, the localization errors of the proposed algorithm in the paper are smaller, indicating that higher localization accuracy has been realized in this paper. This is because the greater the range error is, the higher the requirement for the accuracy of the weight is. In the paper, the proposed algorithm selects the values in accordance with the accuracy of the localization results.

#### 4.1.3. Impact of the Number of Localization Base Stations

**Figure 10:**
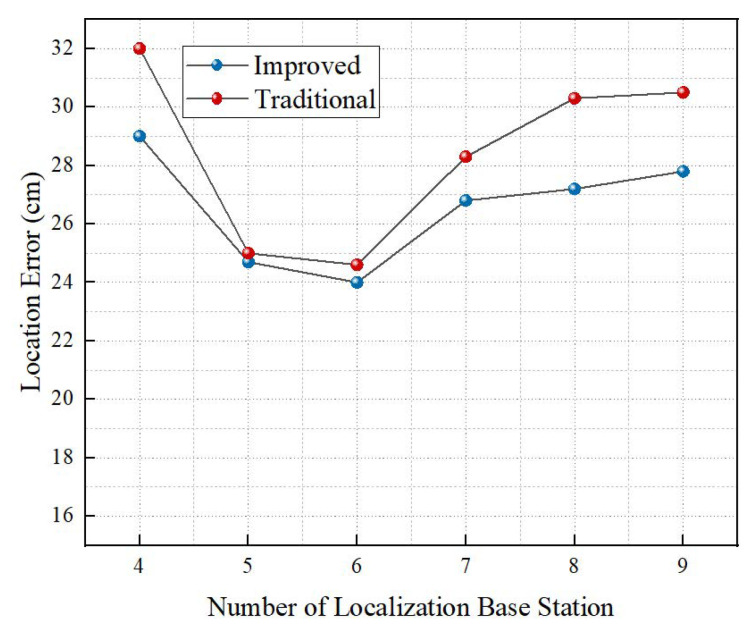
Impact of the number of localization base stations on localization performance.

This section evaluates the effect of the number of base stations participating in multilateral localization on the localization accuracy. In this section, 10 localization base stations are deployed in the simulation area to test the localization accuracy when 4, 5, 6, 7, 8, and 9 base stations are selected, respectively. As can be seen from Figure 10, when the selected base stations participating in multilateral localization are few, the range errors are generally huge and the difference in localization accuracy between the traditional centroid algorithm and the weighted centroid algorithm is small. When the base stations are greater than or equal to 5, the range error is small and the weighted centroid performs better than the traditional centroid. This is because the more reference points there are, the greater the cumulative errors and the higher the requirements for the accuracy of weights are. The improved weighted centroid algorithm chooses the value according to the accuracy of localization results, which avoids the accumulation of errors in the process and leads to higher localization accuracy. Meanwhile, when five or six localization base stations are selected, the localization error is smaller than that of four localization base stations. However, when seven to nine localization base stations are selected, the localization accuracy does not increase but declines due to the excessive introduction of points and more unpredictable errors. Considering the cost of base stations and other factors, the optimal number of base stations is five to six.

### 4.2. Experimental Results

**Figure 11:**
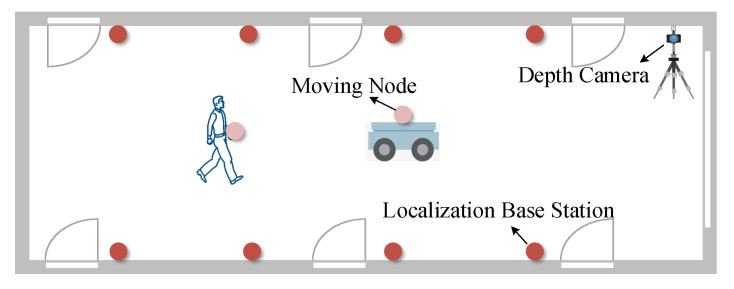
Indoor corridor scene.

**Figure 12:**
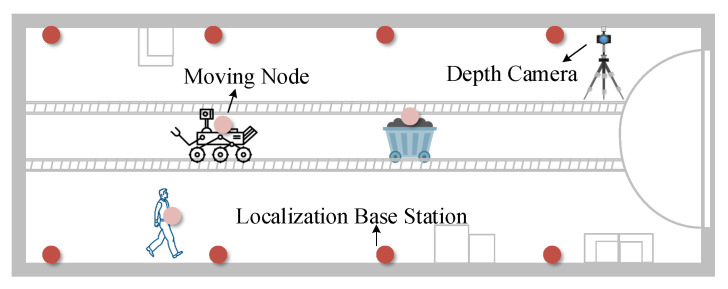
Underground tunnel scene.

As shown in Figure 11 and Figure 12, the MineBL prototype system was constructed in indoor corridors and underground coal mine laboratories, respectively. The localization base stations were placed in the experimental space, and the unknown nodes were placed on the localization targets. We used a D455 camera from the RealSense series, which uses binocular cameras and active infrared imaging. Its maximum resolution of depth is 1280×720, and the furthest detection range is 10 m. Edge computing nodes were introduced to receive and process the camera images and then the location of the robot was estimated by the computers equipped with an Intel Core i5 2.3 GHz CPU and 8 G RAM. Before the experiments, the cameras were calibrated to obtain the internal param needed for localization. In addition, the motion capture system could track the actual location of the robot in real-time. A total of 1000 experiments were performed under each experimental condition to evaluate the overall performance of the system. In addition, the localization performance of the system was closely related to the identification accuracy of nodes and the range accuracy of the depth cameras. The paper also evaluates the influences of various factors, such as ambient light and distance, on identification accuracy and range accuracy.

#### 4.2.1. The Overall Localization Performance

**Figure 13:**
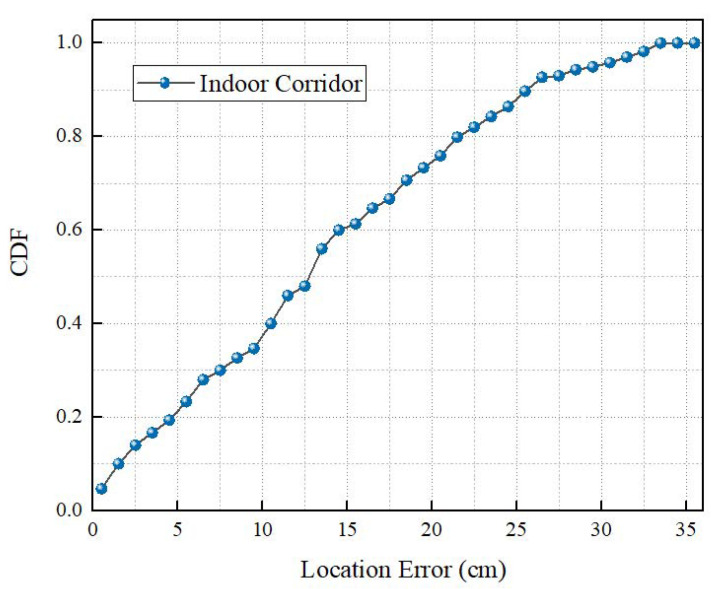
Indoor localization performance.

**Figure 14:**
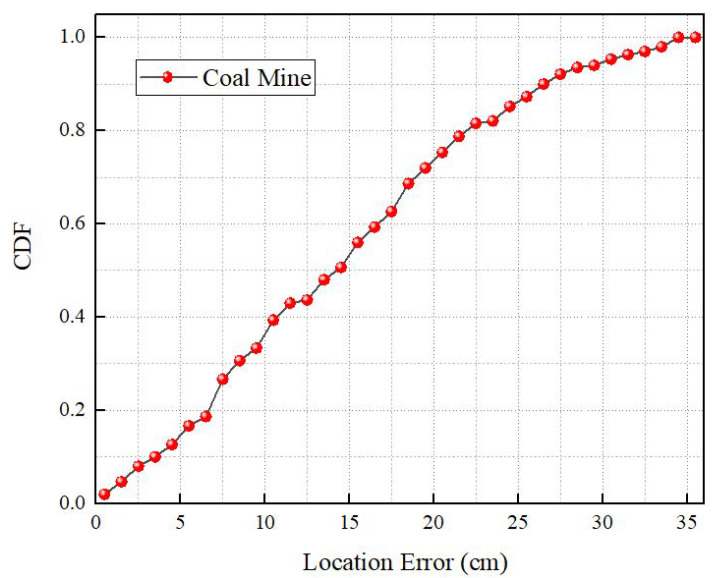
Underground localization performance.

In this section, the overall localization performance of the MineBL is evaluated. Figure 13 and Figure 14 show the cumulative distribution of MineBL localization errors (CDFs) in the indoor corridor and the coal mine underground. It can be seen that in the indoor corridor, the median localization error is about 12.7 cm, and 90% of localization errors are less than 25.6 cm. Moreover, in the coal mine underground, the median localization error is about 14.8 cm, and 90% of localization errors are less than 27.6 cm. The experimental results show that MineBL has good performance in both indoor and coal mine environments. We also evaluated the time required to complete a localization process and found that MineBL only needs 85 ms.

#### 4.2.2. Accuracy of Position Node Recognition

**Figure 15:**
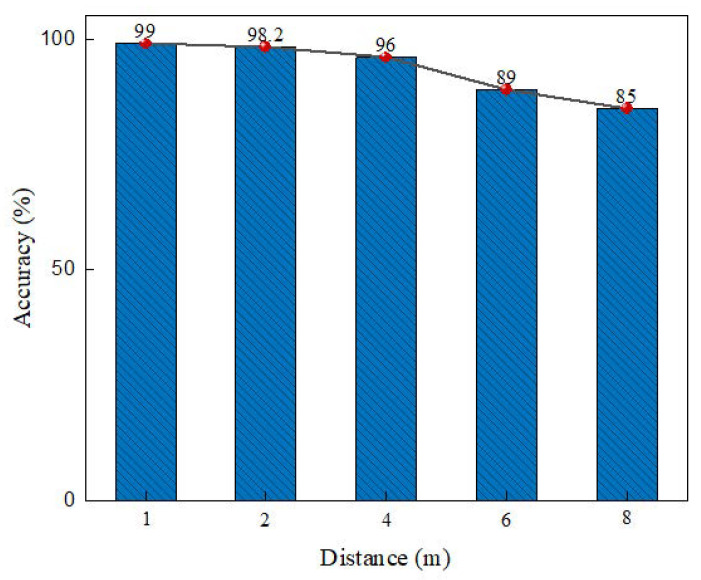
Impact of distance on recognition accuracy.

This section shows the identification accuracy of position nodes under the influences of different distances and diversified ambient light, respectively. The influence of the distance between the nodes and the depth cameras on the identification accuracy of position nodes was evaluated. In the indoor scene, we changed the distance between the nodes and the cameras, and then tested the identification accuracy of the nodes at each distance. As can be seen from Figure 15, within the effective detection range of the depth cameras, the average identification accuracy of nodes decreased with the increase of distance. The average identification accuracy of nodes within 5 m was above 90%, and that of nodes beyond 5 m was above 80%.

**Figure 16:**
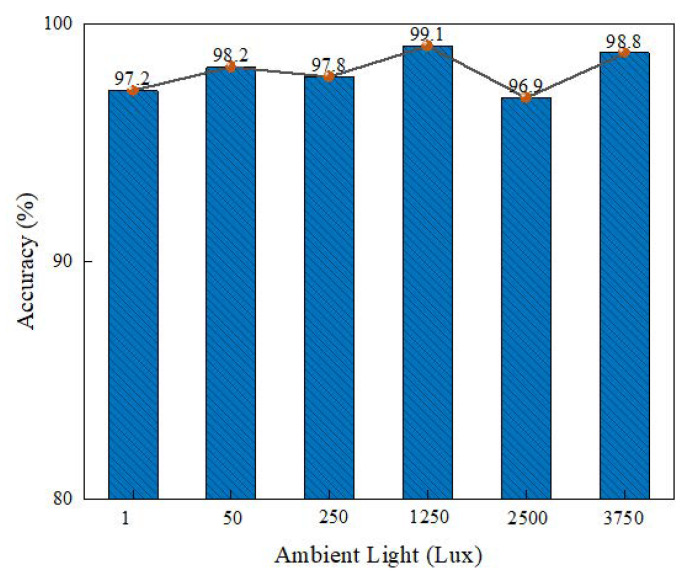
Impact of ambient light on recognition accuracy.

The identification accuracies of nodes under different ambient light intensities were evaluated. The ambient light intensity was changed by turning on and off different numbers of LED lights; seven levels of illumination were selected—1, 50, 250, 1250, 2500, 3750, and 5000—where 1 Lux represents a dark environment. Figure 16 shows that the identification accuracy was kept at about 97% on average, and the change of ambient light had no obvious influence on the identification accuracy under different ambient light intensities.

#### 4.2.3. Accuracy of Ranging

**Figure 17:**
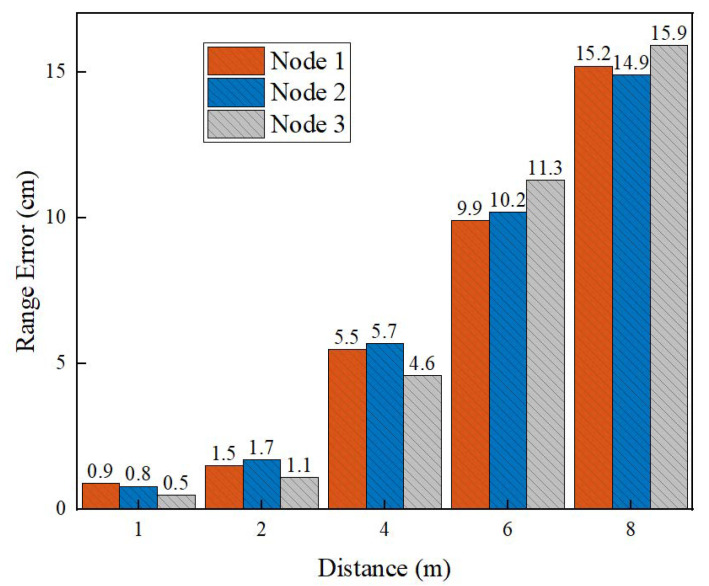
Impact of the distance on ranging accuracy.

Then the influence of the distance between the nodes and the depth cameras on the range accuracy of the system was evaluated. In the indoor scene, we placed three unknown nodes first and tested the range accuracy of the depth cameras at each distance as the distance between the nodes and the cameras changed. As can be seen from Figure 17, within the effective detection range of the depth cameras, the range errors increased with the increase of the distance. The range error within 5 m was less than 10 cm, and the range error within 10 m was no more than 25 cm.

**Figure 18:**
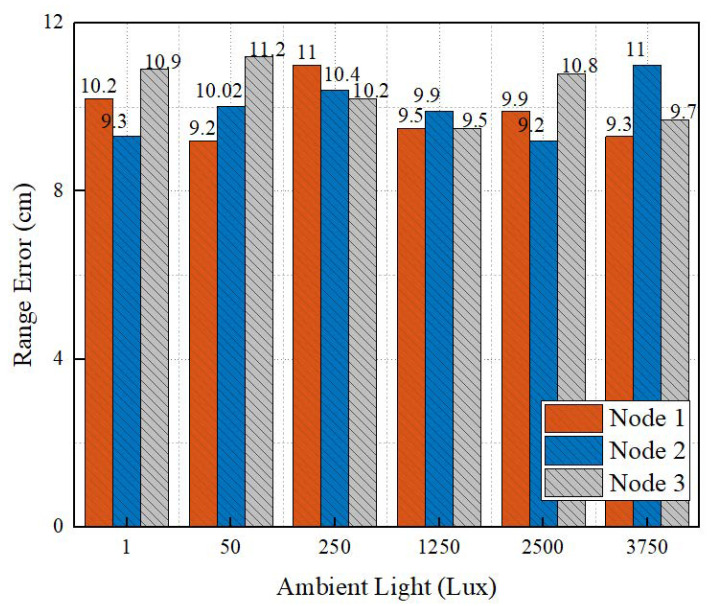
Impact of ambient light on ranging accuracy.

In addition, the range accuracies at different ambient light intensities were evaluated. In the indoor scene, we placed three unknown nodes. The ambient light intensity was changed by turning on and off different numbers of LED lights, and seven levels of illumination were selected—1, 50, 250, 1250, 2500, 3750, and 5000—where 1 lux represents a dark environment. As shown in Figure 18, the influence of ambient light on range errors was not obvious. This is because the multi-filter collaborative denoising method adopted in this paper obtained more accurate depth information and is more robust to the change of ambient light.

## 5. Conclusions

To summarize, a low-cost battery-free localization scheme based on depth images, MineBL, is proposed in this paper; it has good mobility and can migrate with the underground working faces. MineBL uses battery-free and low-cost reflective balls as position nodes to locate underground targets in combination with a series of localization algorithms. Firstly, we designed a data enhancement strategy based on small-target reorganization and further propose a micro-position node recognition algorithm for complex underground coal mine environments. Secondly, we designed a multi-filter cooperative denoising algorithm to obtain the accurate relative distance between the base stations and the targets. Finally, we proposed a weighted centroid location algorithm based on multi-location errors. The weighted factor was determined according to the error of each group of the location data to improve the localization accuracy of the system. A large number of experiments in the indoor laboratory and the underground coal mine laboratory were conducted, respectively, and the experimental results verify that the MineBL underground localization scheme has good localization performances; the localization errors were less than 30 cm in 95% of cases. 

## Data Availability

Not applicable.

## References

[1] Li X., Cao Z., Xu Y. (2021). Characteristics and trends of coal mine safety development. Energy Sources Part A Recover. Util. Environ. Eff..

[2] Xuhui Z., Runlin D., Yongwei L. VR-based remote control system for rescue detection robot in coal mine. Proceedings of the 2017 14th International Conference on Ubiquitous Robots and Ambient Intelligence (URAI).

[3] Wang Y., Tian P., Zhou Y., Chen Q. (2018). The encountered problems and solutions in the development of coal mine rescue robot. J. Robot..

[4] Yang Y., Li Y., Guo X. Underground personnel positioning sysyem based on low-power card reader. Proceedings of the International Conference on Automatic Control and Artificial Intelligence (ACAI 2012).

[5] Szrek J., Zimroz R., Wodecki J., Michalak A., Góralczyk M., Worsa-Kozak M. (2020). Application of the infrared thermography and unmanned ground vehicle for rescue action support in underground mine—The amicos project. Remote Sens..

[6] Babalola O.P., Balyan V. (2021). WiFi fingerprinting indoor localization based on dynamic mode decomposition feature selection with hidden Markov model. Sensors.

[7] Yang Z., Zhou Z., Liu Y. (2013). From RSSI to CSI: Indoor localization via channel response. Acm Comput. Surv..

[8] Chen Z., Zou H., Jiang H., Zhu Q., Soh Y.C., Xie L. (2015). Fusion of WiFi, smartphone sensors and landmarks using the Kalman filter for indoor localization. Sensors.

[9] Zhang T., Zhang K., Liu D., Chen P. CSI-Based Calibration Free Localization with Rotating Antenna for Coal Mine. Proceedings of the International Conference on Wireless Algorithms.

[10] Bargh M.S., de Groote R. Indoor localization based on response rate of bluetooth inquiries. Proceedings of the First ACM international Workshop on Mobile Entity Localization and Tracking in GPS-Less Environments.

[11] Giovanelli D., Farella E., Fontanelli D., Macii D. Bluetooth-based indoor positioning through ToF and RSSI data fusion. Proceedings of the 2018 International Conference on Indoor Positioning and Indoor Navigation (IPIN).

[12] Burzacca P., Mircoli M., Mitolo S., Polzonetti A. “iBeacon” technology that will make possible Internet of Things. Proceedings of the International Conference on Software Intelligence Technologies and Applications & International Conference on Frontiers of Internet of Things 2014.

[13] Li M.G., Zhu H., You S.Z., Tang C.Q. (2020). UWB-based localization system aided with inertial sensor for underground coal mine applications. IEEE Sens. J..

[14] Poulose A., Han D.S. Feature-based deep LSTM network for indoor localization using UWB measurements. Proceedings of the 2021 International Conference on Artificial Intelligence in Information and Communication (ICAIIC).

[15] Seguel F., Palacios-Jativa P., Azurdia-Meza C.A., Krommenacker N., Charpentier P., Soto I. (2022). Underground mine positioning: A review. IEEE Sens. J..

[16] Han Z., Mingxia C., Shunyan L. Research on node location algorithm of Zigbee based on optimized neural network. Proceedings of the 2020 International Conference on Computer Engineering and Application (ICCEA).

[17] Ge Z., Xie L., Wang S., Lu X., Wang C., Zhou G., Lu S. Mag-barcode: Magnet barcode scanning for indoor pedestrian tracking. Proceedings of the 2020 IEEE/ACM 28th International Symposium on Quality of Service (IWQoS).

[18] Fang Y., Cho Y.K., Zhang S., Perez E. (2016). Case study of BIM and cloud-enabled real-time RFID indoor localization for construction management applications. J. Constr. Eng. Manag..

[19] Khattak S., Papachristos C., Alexis K. Vision-depth landmarks and inertial fusion for navigation in degraded visual environments. Proceedings of the International Symposium on Visual Computing.

[20] Ning J. (2011). Indoor object location technology using infrared weaving. Laser Infrared.

[21] Lee C., Chang Y., Park G., Ryu J., Jeong S.G., Park S., Park J.W., Lee H.C., Hong K.s., Lee M.H. Indoor positioning system based on incident angles of infrared emitters. Proceedings of the 30th Annual Conference of IEEE Industrial Electronics Society, IECON 2004.

[22] Iturralde D., Azurdia-Meza C., Krommenacker N., Soto I., Ghassemlooy Z., Becerra N. A new location system for an underground mining environment using visible light communications. Proceedings of the 2014 9th International Symposium on Communication Systems, Networks & Digital Sign (CSNDSP).

[23] Krommenacker N., Vásquez Ó.C., Alfaro M.D., Soto I. A self-adaptive cell-ID positioning system based on visible light communications in underground mines. Proceedings of the 2016 IEEE International Conference on Automatica (ICA-ACCA).

[24] Ali H., Choi J.h. (2019). A review of underground pipeline leakage and sinkhole monitoring methods based on wireless sensor networking. Sustainability.

[25] Thrybom L., Neander J., Hansen E., Landernas K. (2015). Future challenges of positioning in underground mines. IFAC-PapersOnLine.

[26] Guo J., Du J., Xu D. Navigation and positioning system applied in underground driverless vehicle based on IMU. Proceedings of the 2018 International Conference on Robots & Intelligent System (ICRIS).

[27] Cui Y., Liu S., Liu Q. (2021). Navigation and positioning technology in underground coal mines and tunnels: A review. J. S. Afr. Inst. Min. Metall..

[28] Forster C., Carlone L., Dellaert F., Scaramuzza D. (2015). IMU Preintegration on Manifold for Efficient Visual-Inertial Maximum-a-Posteriori Estimation.

[29] Shao W., Vijayarangan S., Li C., Kantor G. Stereo visual inertial lidar simultaneous localization and mapping. Proceedings of the 2019 IEEE/RSJ International Conference on Intelligent Robots and Systems (IROS).

[30] Xu C., Zhang L., Cheng L., Koch R. (2016). Pose estimation from line correspondences: A complete analysis and a series of solutions. IEEE Trans. Pattern Anal. Mach. Intell..

[31] Kanellakis C., Nikolakopoulos G. Evaluation of visual localization systems in underground mining. Proceedings of the 2016 24th Mediterranean Conference on Control and Automation (MED).

[32] Chi H., Zhan K., Shi B. (2012). Automatic guidance of underground mining vehicles using laser sensors. Tunn. Undergr. Space Technol..

[33] Zhang L., Xu C., Lee K.M., Koch R. Robust and efficient pose estimation from line correspondences. Proceedings of the Asian Conference on Computer Vision.

[34] Dang T., Mascarich F., Khattak S., Nguyen H., Khedekar N., Papachristos C., Alexis K. Field-hardened robotic autonomy for subterranean exploration. Proceedings of the 12th Conference on Field and Service Robotics (FSR).

[35] Se S., Lowe D.G., Little J.J. (2005). Vision-based global localization and mapping for mobile robots. IEEE Trans. Robot..

[36] Fauser T., Bruder S., El-Osery A. A comparison of inertial-based navigation algorithms for a low-cost indoor mobile robot. Proceedings of the 2017 12th International Conference on Computer Science and Education (ICCSE).

[37] Huang A.S., Bachrach A., Henry P., Krainin M., Maturana D., Fox D., Roy N., Henrik C.I., Oussama K. (2017). Visual odometry and mapping for autonomous flight using an RGB-D camera. Robotics Research.

[38] Lohar S., Zhu L., Young S., Graf P., Blanton M. (2021). Sensing technology survey for obstacle detection in vegetation. Future Transp..

[39] Ganganath N., Leung H. Mobile robot localization using odometry and kinect sensor. Proceedings of the 2012 IEEE International Conference on Emerging Signal Processing Applications.

[40] Yang S., Hans A., Zhao W., Luo X., Feng C., Rebeca I.G.-B., Liming C., Maria F.C.-U., Chris N. (2020). Indoor localization and human activity tracking with multiple kinect sensors. Smart Assisted Living.

[41] Shorten C., Khoshgoftaar T.M. (2019). A survey on image data augmentation for deep learning. J. Big Data.

[42] Gu K., Tao D., Qiao J.F., Lin W. (2017). Learning a no-reference quality assessment model of enhanced images with big data. IEEE Trans. Neural Networks Learn. Syst..

[43] Tian L., Cao Y., He B., Zhang Y., He C., Li D. (2021). Image enhancement driven by object characteristics and dense feature reuse network for ship target detection in remote sensing imagery. Remote Sens..

[44] Lan W., Dang J., Wang Y., Wang S. Pedestrian detection based on YOLO network model. Proceedings of the 2018 IEEE international conference on mechatronics and automation (ICMA).

[45] Wen H., Dai F., Yuan Y. (2021). A Study of YOLO Algorithm for Target Detection. Adv. Inn Artif. Life Robot..

[46] Ćorović A., Ilić V., Ðurić S., Marijan M., Pavković B. The real-time detection of traffic participants using YOLO algorithm. Proceedings of the 2018 26th Telecommunications Forum (TELFOR).

